# Editorial

**Published:** 2016-07-09

**Authors:** Hasan Özkan

**Affiliations:** Professor, President of Euroasian Gastroenterological Association Editor-in-Chief of EJOHG

**Our Mission and Our Goals**

Dear Colleagues,

Euroasian Gastroenterological Association (EGA) was established on 1996 centered in Ankara, Turkey. Euroasian Gastroenterological Association and our journal are doing very well and getting scientific popularity in the Euroasian Countries year by year. From that day the association has founded many events, including 14 International Congresses in 12 different countries and three Single Topic Conferences (STC) in Europe and Asia.

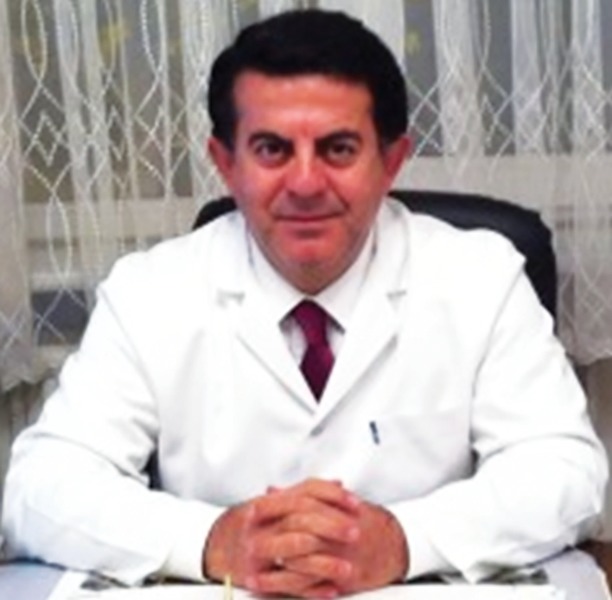



Euroasian Congresses of Gastroenterology and Surgery:

1. 1997 Baku — Azerbaijan
2. 1998 Almati — Kazakhstan
3. 1999 Antalya — Turkey
4. 2000 Tashkent — Uzbekistan
5. 2001 Bishkek — Krygyzhstan
6. 2003 Baku — Azerbaijan
7. 2004 Ankara — Turkey
8. 2005 Tbilisi — Georgia
9. 2006 Baku — Azerbaijan
10. 2007 Isfahan — Iran
11. 2008 Baku — Azerbaijan
12. 2011 Baku — Azerbaijan
  STC 2013 Yalta, Cremea — Ukraine
13. 2013 Baku — Azerbaijan
  STC 2014 Ljubljana — Slovenia
14. 2014 Magosa — Northern Cyprus
  STC 2015 Bucharest — Romania

The official journal of EGA, Euroasian Journal of Hepato-Gastroenterology (EJOHG) is growing rapidly with great efforts by editor-in-chief Hasan Ozkan, co-editor-in-chief Sheikh Mohammad Fazle Akbar, executive editor Mamun-Al-Mahtab, although it was launched only 5 years ago. Euroasian Journal of Hepato-Gastroenterology has began its publishing life in 2011 and it is being published and distributed regularly two times a year. With the big support from our colleagues from Asia and Europe, our journal is continuing its publishing life. In June 2015, we have applied to join the PubMed index which you will be informed about the results. Scientific level of EJOHG has been improving progressively year-by-year with increased number of submitted original articles and review articles.

I have faith that in the coming years our association will grow stronger and will take role in more active affairs. Your support is very important for us. Together, I believe that we can establish a powerful platform and scientific zone in the Eurasia and our dream will come true. Finally, I would like to thank to executive committee, editorial board members and all of my colleagues once again for their valuable support.

**Hasan Özkan**

Professor, President of Euroasian Gastroenterological Association

Editor-in-Chief of EJOHG

